# kNACking on heaven’s door: how important are NAC transcription factors for leaf senescence and Fe/Zn remobilization to seeds?

**DOI:** 10.3389/fpls.2013.00226

**Published:** 2013-07-01

**Authors:** Felipe Klein Ricachenevsky, Paloma Koprovski Menguer, Raul Antonio Sperotto

**Affiliations:** ^1^Centro de Biotecnologia, Universidade Federal do Rio Grande do SulPorto Alegre, Rio Grande do Sul, Brazil; ^2^Departamento de Botânica, Universidade Federal do Rio Grande do SulPorto Alegre, Rio Grande do Sul, Brazil; ^3^Centro de Ciências Biológicas e da Saúde, Programa de Pós-Graduação em Biotecnologia, Centro Universitário UNIVATESLajeado, Rio Grande do Sul, Brazil

**Keywords:** biofortification, iron, NAC transcription factor, nutrient remobilization, senescence, zinc

## Abstract

Senescence is a coordinated process where a plant, or a part of it, engages in programmed cell death to salvage nutrients by remobilizing them to younger tissues or to developing seeds. As Fe and Zn deficiency are the two major nutritional disorders in humans, increased concentration of these nutrients through biofortification in cereal grains is a long-sought goal. Recent evidences point to a link between the onset of leaf senescence and increased Fe and Zn remobilization. In wheat, one member of the NAC (NAM, ATAF, and CUC) transcription factor (TF) family (NAM-B1) has a major role in the process, probably regulating key genes for the early onset of senescence, which results in higher Fe and Zn concentrations in grains. In rice, the most important staple food for nearly half of the world population, the NAM-B1 ortholog does not have the same function. However, other NAC proteins are related to senescence, and could be playing roles on the same remobilization pathway. Thus, these genes are potential tools for biofortification strategies in rice. Here we review the current knowledge on the relationship between senescence, Fe and Zn remobilization and the role of NAC TFs, with special attention to rice. We also propose a working model for OsNAC5, which would act on the regulation of nicotianamine (NA) synthesis and metal–NA remobilization.

## Fe AND Zn BIOFORTIFICATION

Iron (Fe) and zinc (Zn) are essential micronutrients for almost all living organisms and are two of the most versatile metals in biology. Fe participates as a catalytic cofactor in multiple metabolic pathways (photosynthesis, respiration, hormone synthesis, nitrogen fixation, DNA synthesis and repair) due to the ability to participate on reversible redox reactions as Fe^2^^+^ (ferrous) and Fe^3^^+^ (ferric) ions (Puig et al., 2007). Zn does not participate directly on redox reactions, since it occurs in a single oxidation state, but is a key structural component of around 300 enzymes and 2,000 transcription factors (TFs; Palmgren et al., 2008; Prasad, 2012). Both Fe and Zn are present in low quantities in most plant staple foods, leading to Fe and Zn deficiency in humans. Malnutrition of these micronutrients are leading risk factors for disability and death worldwide, especially to children eating cereal-based diets, with low intake of micronutrient-rich foods as meat, poultry, fish, fruits, legumes, and vegetables.

Strategies to alleviate micronutrient malnutrition include fortification (addition during food processing) and supplementation (ingestion of pills or sachets). Although somewhat successful, these approaches are not widely accessible due to logistic and economic issues. A very cost-effective alternative is biofortification, the increase of bioavailable concentrations of an element in edible portions of crops before harvesting (for comprehensive reviews see White and Broadley, 2005; Sperotto et al., 2012a; Carvalho and Vasconcelos, 2013).

Biofortification includes different approaches like soil fertilization or foliar application, conventional breeding and/or transgenic strategies. Mineral fertilization is an effective method to increase seed mineral concentrations, but can be problematic due to continuous cost and environmental carryover. Conventional breeding has been used for decades. Although there is genetic diversity available within existing germplasm collections, rice seems to have the narrowest range, making substantial increases in Fe and Zn concentrations more difficult compared to maize and wheat (Kennedy and Burlingame, 2003; Gómez-Galera et al., 2010; Sperotto et al., 2012a). Thus, it seems imperative that transgenic approaches be used to enable significant increases in Fe and Zn content and bioavailability.

Single or multiple-transgene insertions into the rice genome have successfully increased Fe concentration in grains. Independent over-expression of *OsNAS* genes produced the most promising results so far (Johnson et al., 2011), while other multi-transgene approaches also increased grain Fe concentration (Wirth et al., 2009; Masuda et al., 2012). However, except in the study from Johnson et al. (2011), the levels are still not effective enough to impact human nutrition. An unexplored avenue would be the controlled expression of regulatory genes involved in key processes to Fe and Zn seed allocation. This approach has already been performed to generate rice plants more resistant to Fe deficiency (Kobayashi et al., 2007; Ogo et al., 2011). In order to do that for mineral concentrations in grains, we still need to identify the molecular players relevant to their transport within the plant and during remobilization.

## SENESCENCE PROCESSES: WHAT, WHEN, AND HOW?

Senescence represents endogenously degenerative processes which ultimately lead to organ death. However, it is not a passive process (Yoshida, 2003), but rather a series of coordinated and controlled events. Decline of photosynthesis, chloroplast and chlorophyll degradation, dismantling of biomolecules and decrease in cellular metabolic activities take place, which result in available nutrients and metabolites that can be transported from source (the tissue that supplies nutrients, most commonly green tissues) to sink (the net importer of nutrients, younger or reproductive organs) through the vascular system (Thomas, 2013). Part of leaf senescence seems to be regulated by sugar levels (Rolland et al., 2006; Sperotto et al., 2007), since a senescence-related loss of chlorophyll or protein can be induced by increased sugar contents (Wingler et al., 1998). Hormones and nutrients also contribute to regulation of senescence in source tissues, especially cytokinins, which have a senescence-delaying effect (Sperotto et al., 2009; Davies and Gan, 2012).

The source–sink signaling is not fully understood, and it depends on the species and circumstances. It is already known that senescence in source leaves can be delayed by removal of strong sinks (Zavaleta-Mancera et al., 1999). Elevated levels of N alter sugar signaling in source leaves (Thomas, 2013) and have a significant impact on Fe and Zn acquisition and grain allocation in wheat (Kutman et al., 2011). According to Shi et al. (2012), a sufficient N supply inhibits Fe export from source leaves, but N deficiency enhances Fe pools in source leaves and stimulates Fe export from senescing leaves to sink tissues in barley, corroborating previous findings that high protein concentrations in N-fertilized leaves tend to immobilize Fe and delay senescence (Marschner, 1995).

Nitrogen remobilization from source tissues to seeds (Hortensteiner and Feller, 2002) has received much more attention than metal remobilization over the last decades, since grain yield has been treated as the most important trait to be improved. Part of the seed N is acquired by the roots, but N remobilization from almost all vegetative organs also contributes to the seed N loading, especially during senescence processes (Burstin et al., 2007). In this way, the senescence process partly satisfies grain N concentration, as well with other minerals (Himelblau and Amasino, 2001). However, it is already known that application of fertilizer N generally decreases whole-plant remobilization efficiency (Bahrani et al., 2011).

Previous work has shown that although seed minerals are partially supplied by continuous uptake and translocation during reproductive growth, remobilization of previous stored minerals in green source tissues is also important (Jiang et al., 2007; Waters and Grusak, 2008; Sperotto et al., 2012b), as commonly seen for N. In rice, it was shown that Fe remobilization is dependent on Fe status: under low or sufficient Fe supply, flag leaf Fe remobilization is observed; under high but non-toxic Fe concentrations, there is no Fe remobilization, presumably because of continuous root uptake (Sperotto et al., 2012b). It is also known that mineral remobilization from leaves to seeds can be enhanced by senescence (Zhang et al., 1995; Uauy et al., 2006; Distelfeld et al., 2007; Shi et al., 2012). As several proteins include Fe and Zn ions, a substantial level of metals can be released during leaf senescence due to the high level of protein degradation (Waters et al., 2009).

Recent molecular studies have shown that senescence processes are driven by TF networks that regulate the expression of several senescence-related genes (Guo et al., 2004; Lin and Wu, 2004). One of the most important families of genes described as associated with senescence, and also with nutrient remobilization from source organs to developing seeds, is the NAC (NAM, ATAF, and CUC) family of TFs (Guo et al., 2004; Uauy et al., 2006).

## NAC TRANSCRIPTION FACTORS

Proteins of the NAC family are one of the largest classes of plant-specific TFs. Roles of many NAC TFs have been demonstrated in diverse plant developmental processes. The earliest reports include the NAM (non-apical meristem) protein from petunia (*Petunia hybrida*); *nam* mutants lack the shoot apical meristem (SAM) and die at the seedling stage (Souer et al., 1996). The CUC1/CUC2 (cup-shaped cotyledon) TF from *Arabidopsis*, which participates in the development of embryos and flowers (Aida et al., 1997), defines the boundary domain around organs in the meristem (Nikovics et al., 2006). Later, NAC proteins have been related to diverse processes such as auxin and ethylene signaling (He et al., 2005; Park et al., 2011), cell wall formation (Wang et al., 2011), biotic and abiotic stresses (Puranik et al., 2012), and senescence (Kou et al., 2012).

NAC proteins contain a highly conserved N-terminal domain known as the NAC domain, which has been implicated in DNA binding (Duval et al., 2002; Ernst et al., 2004) as well as protein–protein interactions, forming homodimers or heterodimers with TFs from the NAC family (Ernst et al., 2004; Jeong et al., 2009) or other families (Xie et al., 2000; Greve et al., 2003). The NAC domain reveals a fold consisting of a twisted beta-sheet surrounded by a few helical elements (Ernst et al., 2004). On the other hand, C-terminal regions of NAC proteins are highly divergent (Olsen et al., 2005; Fang et al., 2008), and are related to transcriptional regulation (Xie et al., 2000; Duval et al., 2002).

Many NAC genes have been involved in responses to various environmental stresses like drought, cold, salinity, pathogen attack, and wounding. For recent reviews on NAC TFs in stress response, see Nakashima et al. (2012) and Puranik et al. (2012). In rice, the stress-responsive NAC group (SNAC) includes some already characterized members. *OsNAC5*, *OsNAC6*, and *OsNAC10* are induced by abiotic stresses, abscisic acid (ABA), and methyl jasmonic acid, a plant hormone that activates defense responses against herbivores and pathogens (Ohnishi et al., 2005; Sperotto et al., 2009; Jeong et al., 2010; Takasaki et al., 2010; Song et al., 2011); *OsNAC6* is also induced by biotic stresses (such as wounding and blast disease; Nakashima et al., 2007). Rice plants over-expressing either *OsNAC5*, *OsNAC9*, or *OsNAC10 *under the control of the root-specific RCc3 promoter improved tolerance to abiotic stresses during the vegetative stage of growth and, most importantly, at the reproductive stage, with a concomitant increase in grain yield (Jeong et al., 2010, 2013; Redillas et al., 2012). Another characterized SNAC from rice, OsNAC4, was proposed to lead to hypersensitive response in plants after an appropriate pathogen recognition signal is encountered (Kaneda et al., 2009). In *Arabidopsis*, the SNAC genes *ANAC019*, *ANAC055*, and *ANAC072* are induced by pathogen attack and wounding, and transgenic plants over-expressing either one showed a significant increase in drought tolerance (Tran et al., 2004). Considering that these proteins group with the rice paralogs SNAC1, OsNAC3, OsNAC4, OsNAC5, and OsNAC6, their function seems to be conserved.

Several members of the NAC family have been functionally characterized as playing a prominent role in leaf senescence. In *Arabidopsis*, almost one-fifth of the predicted 109 NAC members are in the database of senescence leaf expression sequence tags (dbEST; Guo et al., 2004). Characterization of NAC TFs involved in senescence processes is also available for other plant species, like rice *OsNAC5* (Sperotto et al., 2009); wheat *NAM-B1* (Uauy et al., 2006); and bamboo *BeNAC1* (Chen et al., 2011). The relation between NAC TFs, senescence and nutrient remobilization will be discussed in the next section.

## SENESCENCE AND METAL REMOBILIZATION: ARE NAC TRANSCRIPTION FACTORS BRIDGING THE GAP?

In monocarpic plants such as wheat and rice, whole-plant senescence is a coordinated process where catabolic activity provides nutrients that are exported and remobilized to developing grains (Matile et al., 1996; Hortensteiner and Feller, 2002). The *Gpc-B1* quantitative trait loci (QTL) from wild emmer wheat (*Triticum turgidum* ssp. *dicoccoides*) was first described as conferring high grain protein content in wheat across diverse environments (Chee et al., 2001; Olmos et al., 2003), an important trait for improving bread and pasta quality. Although already mapped and used in breeding programs, *Gpc-B1* locus was cloned later by Uauy et al. (2006), which found the causal gene to be a NAC TF, NAM-B1. *NAM-B1* expression is up-regulated after anthesis in flag leaves and accelerates senescence. In modern wheat varieties, a 1-bp frame-shift insertion at the *NAM-B1* coding sequence results in a truncated version of the protein, while the wild relative has an intact, fully functional NAM-B1. Silencing of NAM-B1 and other NAM paralogs mimicked the insertion effect, resulting in delayed senescence, decreased grain protein, and lower Fe and Zn concentrations due to reduced nutrient remobilization from vegetative tissues (Uauy et al., 2006; Waters et al., 2009). Moreover, a transcriptomic study showed enrichment of sequences related to transport in wild-type (WT) compared to NAM-B1 RNA interference (RNAi) lines during senescence (Cantu et al., 2011). Taken together, these results established NAM-B1 as a positive regulator of senescence and nutrient remobilization during grain maturation, suggesting that an early senescence onset could lead to increased Fe and Zn concentrations in grains (Uauy et al., 2006).

To describe genes with similar functions in other crops, an obvious avenue would be looking at orthologous proteins. The closest homolog of NAM-B1 in the rice genome, named ONAC010 (LOC_Os07g3792; Uauy et al., 2006), has a function in flower development but not in senescence, as neither ONAC010 loss-of-function nor over-expression have the expected effects on senescence timing (Distelfeld et al., 2012). Thus, another paralog with lower sequence similarity could play the NAM-B1 role in rice plants. In this context, *OsNAC5* was demonstrated to be a senescence associated gene that is up-regulated during grain maturation in rice flag leaves (Sperotto et al., 2009). *OsNAC5* is regulated by ABA, a hormone with a known central role in senescence processes (Lim et al., 2007). A comparison of diverse cultivars showed a positive correlation of *OsNAC5* expression in flag leaves before and during anthesis with final Fe, Zn, and protein concentrations in mature grains (Sperotto et al., 2009, 2010). These results showed that *OsNAC5 *expression pattern resembles that of *NAM-B1*, and suggested that OsNAC5 could act during senescence-associated nutrient remobilization to rice grains, probably downstream on the senescence onset pathway.

Transgenic plants bearing constructs with *OsNAC5* under the control of a constitutive or a root-specific promoter were generated in a recent work. When OsNAC5 was expressed only in roots, plants increased root diameter and improved recovery after drought stress (Jeong et al., 2013). Interestingly, transcriptomic analysis of roots from both transgenic lines showed commonly up-regulated genes, indicating potential targets of OsNAC5, but not necessarily linked to changes in root morphology (Jeong et al., 2013). Among them, *Nicotianamine Synthase 2* (*OsNAS2*) and *Yellow Stripe-Like 2* (*OsYSL2*), two genes related to metal homeostasis, were up-regulated. OsNAS2 is a key enzyme in nicotianamine (NA) synthesis, a low-molecular weight compound that chelates metals and a precursor for phytosiderophore synthesis (Higuchi et al., 1999), while OsYSL2 is an Fe–NA transporter expressed in phloem cells (Koike et al., 2004). Both NA and OsYSL2 are involved in Fe seed loading and metal long distance transport through the phloem (Koike et al., 2004; Curie et al., 2009; Klatte et al., 2009; Ishimaru et al., 2010; Schuler et al., 2012).

Different studies point that regulation of NA synthesis and metal–NA complex transporters could be involved in remobilization. Constitutive over-expression or activation-tagging of *OsNAS1*, *OsNAS2*, or *OsNAS3* genes in rice increased concentrations of Fe, Zn, or both in grains (Lee et al., 2009, 2011, 2012; Johnson et al., 2011). Concomitant insertion of constructs driving *Hordeum vulgare NAS1* constitutive expression, *OsYSL2* expression in phloem cells and endosperm and *Ferritin* in endosperm, led to increased Fe concentrations (and Zn to a lower extent) in grains (Masuda et al., 2012). In *H. vulgare*, both dark and N deficiency-induced senescence up-regulated *HvNAS2* expression in leaves, resulting in increased phytosiderophore concentration rather than NA (Shi et al., 2012). In *A. thaliana*, loss-of-function of *OsYSL2* homologs, *AtYSL1* and *AtYSL3*, resulted in reduced remobilization of metals to seeds during senescence (Waters et al., 2006).

**FIGURE 1:**
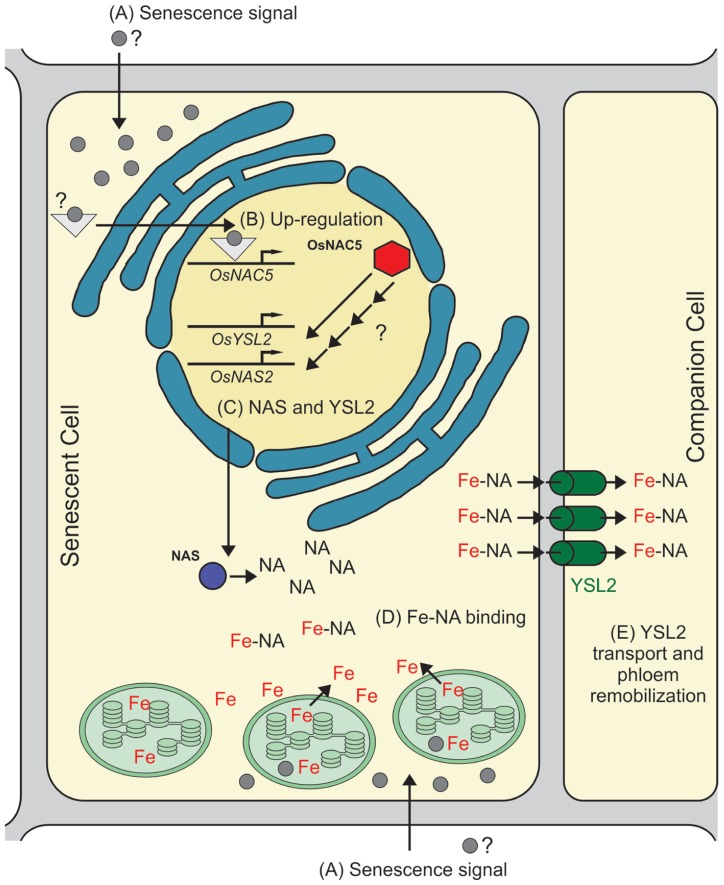
**Proposed model for OsNAC5 role in senescence and metal remobilization.** The model is based on indirect evidence provided by several studies, especially on Sperotto et al. (2009) and Jeong et al. (2013), and shows only Fe remobilization, although a similar pathway is likely to be involved in Zn and other metals remobilization. **(A)** A signal is sensed by the cell, triggering the senescence-associated cellular components degradation, including chloroplasts, the main site of Fe concentration in the cell. **(B)** *OsNAC5* transcription is up-regulated by the senescence downstream signaling pathway. **(C)** OsNAC5 protein is produced and triggers the up-regulation of *OsNAS2* and *OsYSL2* transcription (based on microarray data presented in the work performed by Jeong et al., 2013). The increased transcription observed is either directly or indirectly regulated by OsNAC5. **(D)** OsNAS2 protein increases intracellular NA concentration, which in turn chelates free Fe coming from chloroplast and other cellular components degradation. **(E)** The Fe–NA complex is transported across the plasma membrane of the senescent cell, and then transported into phloem by OsYSL2, which allows Fe–NA complex long distance translocation. It is important to point the possibility that Fe and NA are exported independently from the cell, interacting in the apoplast and then transported into the phloem by OsYSL2.

Therefore, we speculate that OsNAC5 has a role in senescence and metal movement to grains by controlling, either directly or indirectly, the biosynthesis of NA and metal transport through the phloem. Our proposed model, based on data from the literature, is shown in **Figure 1**: (A) a senescence signal is sensed by the cell, activating signaling molecules that regulate the onset of senescence; (B) *OsNAC5 *transcription is up-regulated as part of the senescence-induced nutrient remobilization process; (C) OsNAC5 protein up-regulates, either directly or indirectly, *OsNAS2* and *OsYSL2 *transcription, as well as other targets (not necessarily related to metal remobilization); (D) OsNAS2 increases NA production, which binds free Fe coming from cellular degradation; (E) after efflux from the cell, OsYSL2 acquires the Fe–NA complex into phloem cells for long distance transport. It is important to note that, while OsYSL2 was not demonstrated to transport Zn–NA complexes, NA is able to bind Zn^2^^+^, and Zn–NA complexes are the major Zn form found in the rice phloem sap (Nishiyama et al., 2012). Thus, other transporters could be playing a similar role to OsYSL2 to load Zn–NA into the phloem.

However, work analyzing distinct *OsNAC5* over-expressing lines did not show up-regulation of *OsNAS2* or *OsYSL2* (Takasaki et al., 2010). We should consider that the increased expression observed by Jeong et al. (2013) was a result from roots transcriptomic analyses, which is not the tissue where remobilization takes place. Moreover, up-regulation of these genes could be an indirect effect rather than a result of OsNAC5 binding to *OsNAS2* or *OsYSL2* promoters.

Transcriptomic analysis of *NAM-B1 *RNAi wheat lines did not reveal down-regulation of homologous sequences to *NAS2* or *YSL2 *compared to WT, but rather putative *ZIP* (zinc-regulated/iron-regulated transporter) and *NRAMP* (natural resistance associated macrophage protein) metal transporters (Cantu et al., 2011), indicating that NAS and YSL homologs are not regulated by NAM-B1. This could be due to the fact that NAM-B1 and OsNAC5 are not necessarily regulating the same set of genes. NAM-B1 silencing leads to late senescence, while the functional version from wheat wild relative accelerates it. On the other hand, OsNAC5 over-expressing plants were not reported as senescing earlier than WT (Takasaki et al., 2010; Song et al., 2011; Jeong et al., 2013), indicating that OsNAC5 increased expression cannot trigger senescence alone. Silencing of *OsNAC5* did not lead to late senescence as well (Song et al., 2011). OsNAC5 is known to homo- and heterodimerize with other TFs (Jeong et al., 2009), which could be necessary for OsNAC5-mediated response. Thus, it seems that OsNAC5 is acting more downstream on the senescence pathway than NAM-B1, or even in a distinct parallel regulatory network, regulating a different set of senescence-related processes.

Although we should be cautious in the analysis of the available evidence, our model seems to be holding as the first attempt to point out the mechanism of NAC proteins in mineral remobilization in crops. Further work will be necessary to clearly elucidate the role of OsNAC5 in senescence and metal remobilization, as well to functionally demonstrate which genes are controlled by this TF. If true, the regulation of metal remobilization by OsNAC5 could be an interesting avenue for biofortification strategies.

## Conflict of Interest Statement

The authors declare that the research was conducted in the absence of any commercial or financial relationships that could be construed as a potential conflict of interest.
